# Building an intelligent diabetes Q&A system with knowledge graphs and large language models

**DOI:** 10.3389/fpubh.2025.1540946

**Published:** 2025-02-20

**Authors:** Zhenkai Qin, Dongze Wu, Zhidong Zang, Xiaolong Chen, Hongfeng Zhang, Cora Un In Wong

**Affiliations:** ^1^School of Information Technology, Guangxi Police College, Nanning, China; ^2^School of Computer Science and Artificial Intelligence, Southwest Jiaotong University, Chengdu, China; ^3^School of Social Development, Yangzhou University, Yangzhou, China; ^4^Faculty of Humanities and Social Sciences, Macao Polytechnic University, Macao, China

**Keywords:** knowledge graph, Q&A system, large language models, prompt learning, personalized health management

## Abstract

**Introduction:**

This paper introduces an intelligent question-answering system designed to deliver personalized medical information to diabetic patients. By integrating large language models with knowledge graphs, the system aims to provide more accurate and contextually relevant medical guidance, addressing the limitations of traditional healthcare systems in handling complex medical queries.

**Methods:**

The system combines a Neo4j-based knowledge graph with the Baichuan2-13B and Qwen2.5-7B models. To enhance performance, Low-Rank Adaptation (LoRA) and prompt-based learning techniques are applied. These methods improve the system's semantic understanding and ability to generate high-quality responses. The system's performance is evaluated using entity recognition and intent classification tasks.

**Results:**

The system achieves 85.91% precision in entity recognition and 88.55% precision in intent classification. The integration of a structured knowledge graph significantly improves the system's accuracy and clinical relevance, enhancing its ability to provide personalized medical responses for diabetes management.

**Discussion:**

This study demonstrates the effectiveness of integrating large language models with structured knowledge graphs to improve medical question-answering systems. The proposed approach offers a promising framework for advancing diabetes management and other healthcare applications, providing a solid foundation for future personalized healthcare interventions.

## 1 Introduction

Diabetes has become a significant global health challenge, with its prevalence rising dramatically in recent years. According to the World Health Organization, the number of individuals diagnosed with diabetes increased from 150 million in 2000 to 537 million in 2021 (1). The associated complications of diabetes not only increase mortality rates, but also amplify the overall burden of the disease, emphasizing the urgent need for continuous monitoring of blood glucose and timely and effective treatment options. Despite the availability of numerous medical data sources, patients often struggle to access accurate and personalized information. Existing diabetes management platforms, such as MySugr and BlueLoop, have made notable strides in glucose monitoring; however, they face limitations in managing complex patient conditions (2–5). Consequently, their effectiveness in dynamic healthcare environments is constrained.

Recent advancements in artificial intelligence (AI) have fueled the development of intelligent question-answering (QA) systems that leverage large language models (LLMs) to improve access to medical information. Despite these advances, substantial challenges remain, particularly in achieving deep semantic understanding and generating personalized responses in complex medical contexts (6). Diabetes-related health information is inherently multifaceted, encompassing areas such as pathogenesis, diagnostic metrics, treatment protocols, and potential complications. As a result, effective contextual comprehension and integration of diverse knowledge sources present significant challenges for traditional QA systems, limiting their adaptability to patients' evolving needs (7).

Knowledge graphs offer a structured approach to managing large volumes of medical information, enabling efficient organization, representation, and retrieval of healthcare data. Building a diabetes-specific knowledge graph facilitates rapid retrieval of relevant entities and relationships, logical reasoning, information integration, and the provision of personalized, accurate responses. Integrating a Neo4j-based knowledge graph with a large language model significantly enhances the system's ability to retrieve and synthesize information, thereby improving its capacity to handle multifaceted inquiries and support sophisticated clinical reasoning (8).

This study introduces an innovative diabetes-focused question-answering system that integrates a knowledge graph with a large language model to enhance personalized information retrieval and address the limitations of traditional medical QA systems. By combining structured medical knowledge from the knowledge graph with the language processing power of a large language model, the system enhances its understanding of complex medical scenarios, thereby improving response accuracy and depth. The following sections provide a comprehensive overview of the technical background, system architecture, experimental results, and future research directions, highlighting the potential applications of intelligent QA systems in healthcare. The key contributions of this study include the following:

1. Improved accuracy in recognizing essential entities and identifying user intents within diabetes-related intelligent QA systems.
2. Effective integration of intent detection and entity recognition to enhance knowledge graph querying capabilities.
3. Optimization of knowledge graph and large language model integration to provide personalized medical recommendations.

## 2 Related work

### 2.1 Named entity recognition

Named Entity Recognition (NER) is a foundational task in natural language processing (NLP) that has evolved from rule-based and statistical methods to more sophisticated approaches involving deep learning and pre-trained models. Early NER methods relied heavily on rule-based systems and statistical models, such as Conditional Random Fields (CRF) and Hidden Markov Models (HMM). Lafferty et al. demonstrated the effectiveness of CRF for sequence labeling, although its reliance on handcrafted features limited scalability across diverse and complex domains (9). Similarly, Rabiner's work on HMMs proved useful for sequence data but suffered from significant limitations, including a dependency on domain-specific knowledge, reducing its general applicability.

The advent of deep learning marked a major shift in NER research, moving toward more advanced neural network-based approaches. Huang et al. were among the first to utilize Bidirectional Long Short-Term Memory Networks (BiLSTM) for NER, demonstrating their capacity to effectively capture long-range dependencies (10). Subsequently, Ma and Hovy enhanced sequence labeling performance by integrating Convolutional Neural Networks (CNN) with LSTM, showcasing a hybrid architecture that outperformed earlier NER models (11). Despite these advancements, deep learning models often struggled with capturing global semantic context, focusing primarily on local contextual features, which limited their ability to fully interpret complex sequences (12).

Transformer-based models have fundamentally transformed NER. Devlin et al. introduced the Bidirectional Encoder Representations from Transformers (BERT) model, which significantly improved NER performance through large-scale pre-training and transfer learning (13). Liu further expanded this work with RoBERTa, enhancing contextual understanding by removing the next-sentence prediction task and significantly increasing the amount of training data and training duration (14). However, even Transformer models face substantial challenges in specialized domains, such as medicine and law, where pre-training data may not adequately capture domain-specific terminology or complex linguistic structures (15). For example, Lee et al. demonstrated that general-purpose BERT models often underperform in medical contexts compared to specialized models like BioBERT, which was specifically fine-tuned using medical-domain data, significantly outperforming general models on medical-related tasks (16).

To address these challenges, recent innovations have emerged. Thaminkaew et al. introduced Prompt-based Learning, which employs carefully crafted prompts to direct the model's attention to specific tasks, thus reducing the need for extensive annotated datasets, particularly in low-resource environments (17). Additionally, Hu et al. proposed the Low-Rank Adaptation (LoRA) technique, which fine-tunes model parameters using low-rank matrix decomposition, effectively lowering computational costs while improving NER performance (18).

Despite substantial progress, significant challenges remain in NER. Traditional models rely heavily on domain expertise and extensive feature engineering, limiting adaptability to varied text scenarios. Although deep learning has significantly advanced performance, these models often still struggle with global semantic comprehension and domain-specific adaptation. Transformer-based models, while excelling in general tasks, often fail to handle specialized terminologies effectively, particularly in domains like medicine and law. To address these issues, this study proposes an NER approach that integrates Prompt-based Learning with LoRA fine-tuning, aiming to improve adaptability and accuracy in specialized domains, especially under low-resource conditions. This strategy aims to enhance both the precision and robustness of NER in such fields, minimizing dependence on large annotated datasets.

### 2.2 Knowledge graphs

Knowledge graphs provide a structured framework for organizing and representing complex medical information, thereby facilitating efficient retrieval and supporting advanced analytics. In the healthcare domain, knowledge graphs have demonstrated significant potential for integrating diverse medical information sources. Ji et al. developed knowledge graph embedding models that enable collective learning over multi-relational data, laying the foundation for more effective knowledge representation (19). Yu et al. introduced MedGraph, a comprehensive medical knowledge graph that integrates diagnostic information, drug data, and patient history, thereby enhancing diagnostic accuracy (20). Moreover, Zhang and Lee employed Neo4j to connect patient descriptions with existing medical knowledge, resulting in a symptom-based disease query system that improved query precision and relevance.

Despite these advancements, healthcare knowledge graphs still face significant challenges, such as integrating heterogeneous data sources and ensuring real-time updates of medical knowledge. The effectiveness of these systems largely depends on their ability to represent intricate medical relationships in a manner that can be effectively leveraged by downstream applications. Additionally, the adaptability and scalability of existing knowledge graphs within dynamic healthcare environments remain limited.

To address these challenges, recent studies have explored integrating knowledge graphs with predictive analytics to enhance clinical decision-making. For instance, Gupta et al. constructed a predictive healthcare knowledge graph to anticipate patient health trajectories by linking clinical records with biomedical research data. Liu and Thompson suggested that future research should focus on developing scalable and flexible automated data integration solutions to improve adaptability in dynamic healthcare settings (21).

### 2.3 Question-answering system

Recent advances in question-answering (QA) systems have greatly benefited from the integration of large-language models (LLMs), leading to substantial improvements in response precision and coherence. Harnoune et al. proposed a hybrid model that combines BERT with a knowledge graph, enhancing intent recognition accuracy within medical QA systems (22). However, LLMs often lack the domain-specific understanding required to effectively address specialized healthcare queries. Chen et al. highlighted that general pre-trained models frequently provide incomplete or inaccurate responses to professional medical inquiries, especially for personalized, patient-specific questions (23). Bommasani et al. further emphasized that, while foundation models present significant opportunities for QA systems, they carry inherent risks related to domain-specific inadequacies and biases, particularly concerning sensitive fields like healthcare (24).

Recent research has attempted to overcome these limitations by integrating LLMs with knowledge graphs, thereby improving domain-specific comprehension and enhancing QA coherence. Santos et al. demonstrated that incorporating contextual information from knowledge graphs into Transformer-based models significantly improved their ability to handle complex medical dialogues. Nevertheless, current systems still struggle to maintain coherence in multi-turn dialogues and effectively integrate structured knowledge graph data with the generative capabilities of LLMs (25).

Another promising line of research involves prompt-based learning approaches to improve QA system performance in healthcare. By designing specific prompts to direct LLMs to generate more precise and contextually relevant responses, prompt-based methods significantly enhance the models' ability to comprehend nuanced medical queries. Hu et al. employed a prompt-based approach integrated with a medical knowledge graph to effectively identify key entities in patient questions, subsequently improving the precision of follow-up recommendations (26). These methods are particularly advantageous in addressing the domain-specific gaps often exhibited by LLMs when dealing with specialized healthcare content.

### 2.4 Limitations and proposed approach

Based on this analysis, several limitations remain in the integration of knowledge graphs with intelligent question-answering (QA) systems. First, a disconnect between intent recognition and knowledge retrieval often undermines response coherence and accuracy. Second, the accuracy of current models requires further improvement to effectively manage complex medical queries. Additionally, current systems need enhanced capabilities to manage multi-turn dialogues and personalize the processing of patient information.

To address these challenges, this study proposes a diabetes-focused QA system that integrates knowledge graphs with large language models (LLMs). The proposed system improves interactions between intent recognition, entity recognition, and knowledge retrieval to provide more precise and personalized health advice. Initially, the system employs a pretrained language model to perform semantic analysis of patient queries, identifying intents and extracting key entities through a fine-tuned model. During knowledge retrieval, the Neo4j database is queried for relevant medical information, which is then used to generate natural language responses through the LLM. This approach aims to improve the precision and professionalism of responses while improving the system's ability to address personalized patient needs, thus supporting diabetes patients in self-management and treatment decision-making (27).

## 3 Method

### 3.1 Research framework

**Figure 1:**
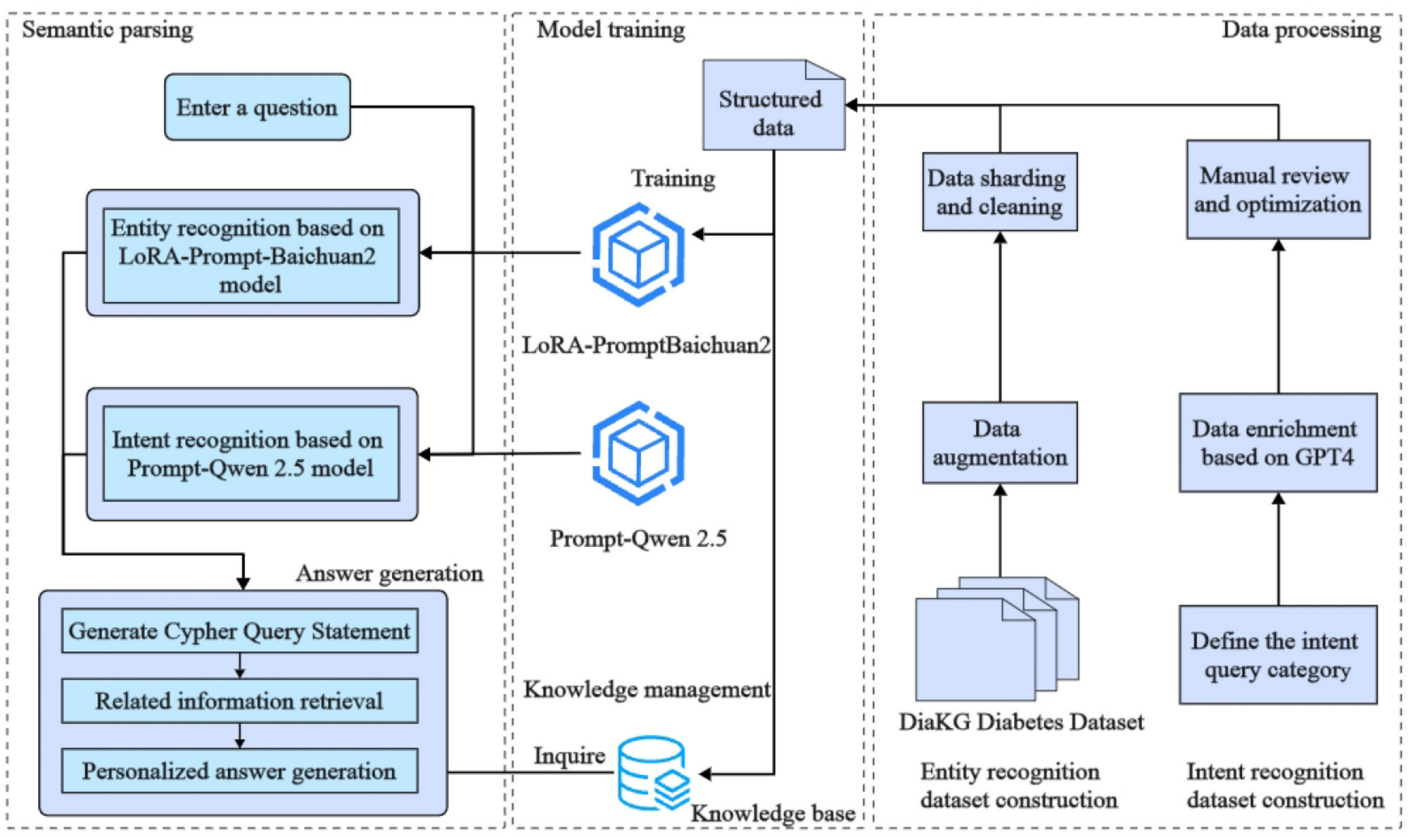
The architecture of the diabetes intelligent question-answering (QA) system. The diagram shows the integration of entity recognition, intent recognition, and knowledge graph querying components. These components work together to generate tailored responses for diabetes-related inquiries, providing more precise and personalized answers.

The aim of this study is to design and implement an efficient intelligent question-answering (QA) system for diabetes, addressing current limitations in medical entity recognition and user intent comprehension. These limitations include challenges such as low accuracy in entity extraction, limited scalability across different medical scenarios, and the restricted availability of relevant data. The proposed system architecture, illustrated in Figure 1, integrates the Baichuan2-13B large language model with Low-Rank Adaptation (LoRA) fine-tuning techniques and prompt-based learning. The architecture focuses on accurately identifying key medical entities, such as disease categories, examination indicators, and medication names, in order to enhance system responsiveness and precision.

The Baichuan2-13B model is utilized for medical entity identification within the input text. The use of LoRA reduces resource requirements during model fine-tuning, which allows efficient adaptation to new domains. Furthermore, prompt-based learning is employed to guide the model in identifying specific entities using explicit instructions, such as focusing on disease types or particular examination indicators. To improve the model's generalizability across various contexts, data augmentation techniques are also applied to enrich the training dataset.

In addition, the system integrates prompt learning with large language models to recognize user intents, such as requests for disease overviews, symptoms, causes, and related medical information. After recognizing entities and intents, the system formulates queries to extract relevant medical knowledge from a diabetes-specific knowledge graph, which includes details such as disease overviews, preventive measures, medications, and examination indicators. Finally, a large language model is used to generate context-specific responses, ensuring both personalization and precision in the medical information provided. Overall, the proposed framework demonstrates substantial potential for improving the accuracy and efficiency of question-answering systems in medical contexts.

### 3.2 Named entity recognition method

This study advances Named Entity Recognition (NER) for diabetes-related texts by leveraging Low-Rank Adaptation (LoRA) in conjunction with prompt-based learning techniques. These combined approaches facilitate efficient model adaptation and precise entity recognition, even in low-resource environments, such as those with limited computational resources or small datasets. Detailed information about the cue design and fine-tuning process is shown later in the article.

#### 3.2.1 Prompt template design

The proposed system integrates LoRA fine-tuning with prompt-based learning by utilizing the Baichuan2-13B model to enhance entity recognition. LoRA allows the model to sustain high efficiency while significantly reducing hardware requirements, thereby enabling flexible adaptation to new domain data. Simultaneously, prompt-based learning provides explicit task instructions, guiding the model to adopt a targeted approach toward specific goals and ensuring accurate identification of critical entities.

The proposed system integrates prompt-based learning to guide the large language model in identifying specific diabetes-related entities. The prompt template is designed to instruct the model to extract relevant medical terms based on a predefined schema. An example of the prompt is: “You are an expert in diabetes entity extraction. Extract entities such as ‘symptoms', ‘treatment methods', and ‘medications' from the input and return an empty list for any nonexistent entities. The result should be in JSON format, with the following categories: ‘symptoms', ‘treatment methods', ‘medications', etc.” This explicit task definition enables the model to focus on domain-specific categories, improving its ability to recognize critical entities in medical texts. The schema encompasses specific entity categories, such as “pathogenesis,” “non-drug treatment,” “surgery,” “examination indicators,” “body parts,” and “clinical manifestations,” among others. An example of the prompt template is presented in Table 1.

**Table 1:** Description of fields used in named entity recognition task.

**Field**	**Element**
Instruction	The prompt that provides the task instructions required by the model. It specifically includes extracting entities from diabetes-related texts and returning the results in JSON format.
Schema	Definition of entity categories, including “Medication Name”, “Examination Indicator”, “Cause”, “Duration”, “Examination Indicator Value”, “Pathogenesis”.
Input	Input text, which needs entity recognition. For example: “Routine monitoring of vitamin B12 levels is not recommended for patients taking metformin.”
Output	Model output, where entities are identified and extracted from the input text and returned in JSON format as per the prompt requirements.

To formalize the prompt-based learning approach, let *P* be the prompt and *X* be the input text. The concatenated input to the model can be expressed, as shown in Equation 1:


(1) $$\begin{array}{l} X^{\prime }=\left[P;X\right] \end{array}$$

where [*P*; *X*] represents the concatenation of the prompt *P* and the input text *X*. This concatenation ensures that the model is explicitly guided by the prompt, thereby focusing on extracting relevant entities according to the schema definition.

The output of the model is a sequence of entity predictions *y* = (*y*_1_, *y*_2_, …, *y*_*n*_), where each *y*_*i*_ represents the prediction for a particular token in the input sequence. The objective of prompt-based learning is to maximize the probability of correctly predicting each entity, as shown in Equation 2:


(2) $$\begin{array}{l} ŷ=\arg\underset{y}{\max}P\left(y|X^{\prime },\theta \right) \end{array}$$

where θ represents the model parameters. The goal is to optimize the conditional probability *P*(*y*|*X*′, θ) given the prompt-enhanced input *X*′.

The prompt-based strategy effectively guides the Baichuan2-13B model in sequentially assessing text content, ensuring adherence to predefined entity categories and facilitating the extraction of relevant information. When combined with Low-Rank Adaptation (LoRA) fine-tuning, the model demonstrates rapid adaptability while preserving high accuracy in recognizing complex medical entities, even in resource-constrained or limited-data scenarios. The strength of this approach lies in its capacity to accurately identify key entities through explicit task instructions, thereby reducing errors and omissions (18).

#### 3.2.2 LoRA fine-tuning

**Figure 2:**
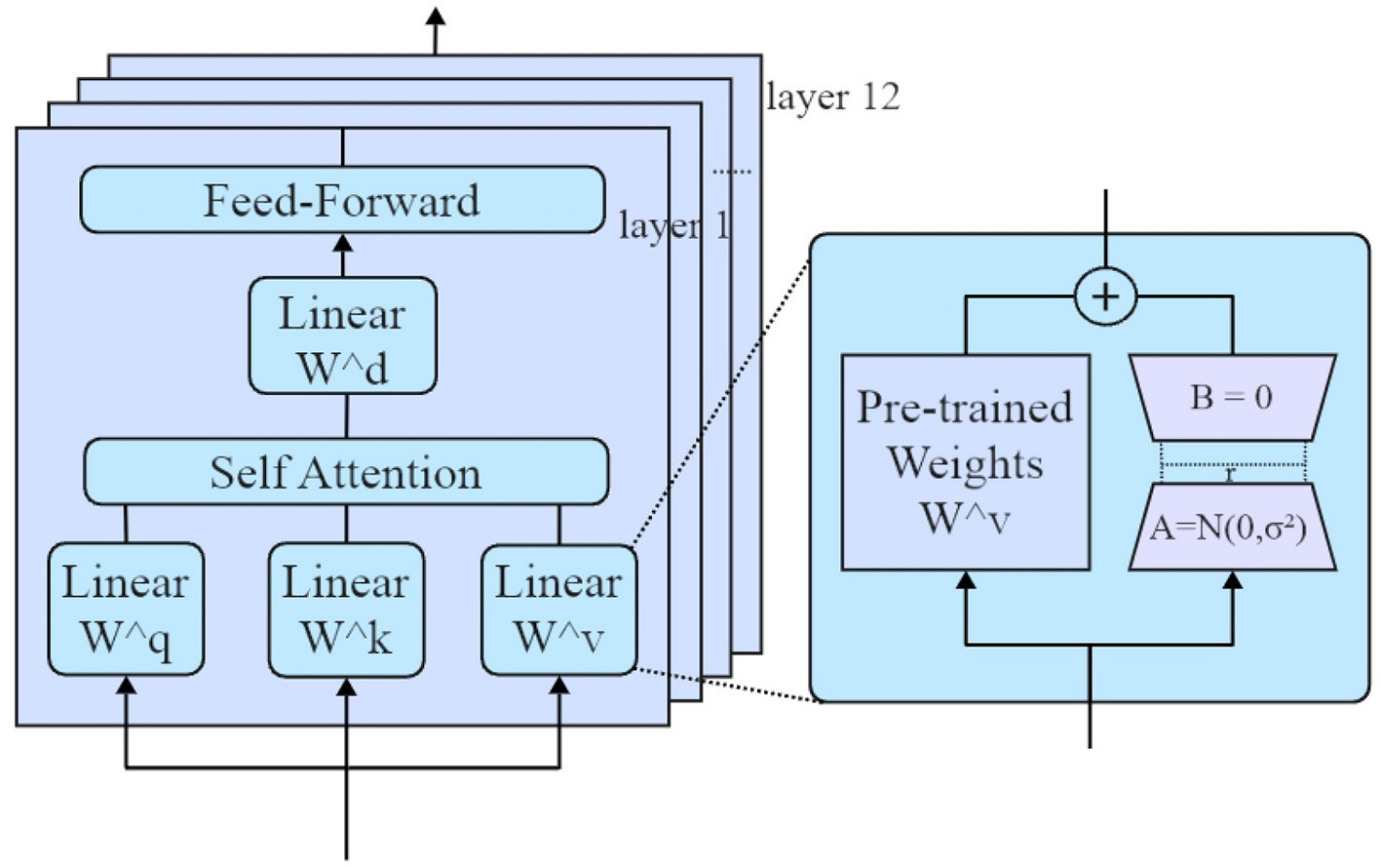
The architecture of the Low-Rank Adaptation (LoRA) fine-tuning process, which efficiently adapts transformer models by approximating their weight matrices with low-rank representations. The model comprises twelve transformer layers, each integrating a self-attention mechanism and feed-forward networks. LoRA targets the fine-tuning of the value weight matrix *W*^*v*^ by decomposing it into two low-rank matrices *A* and *B*, reducing computational costs and memory requirements.

Low-Rank Adaptation (LoRA) is an efficient parameter fine-tuning technique that approximates the model's weight matrix using low-rank matrices, thereby significantly reducing computational and memory requirements during the fine-tuning process. The architecture of the LoRA Fine-Tuning process is illustrated in Figure 2.

Specifically, LoRA approximates the model's weight matrix *W* as follows, as shown in Equation 3:


(3) $$\begin{array}{l} W^{\prime }=W+\Delta W,\;\Delta W=AB \end{array}$$

where *A* ∈ ℝ^*d*×*r*^ and *B* ∈ ℝ^*r*×*k*^ are low-rank matrices, with *r* ≪ min(*d, k*). This low-rank decomposition effectively reduces the number of trainable parameters, making the adaptation process computationally efficient.

In this study, LoRA was used to fine-tune the Baichuan2 model specifically for diabetes-related medical texts. Leveraging LoRA allows the model to maintain high efficiency while substantially reducing hardware resource demands. This optimization facilitates flexible adaptation to new domain-specific data, thereby improving entity recognition accuracy.

To optimize the model during fine-tuning, the cross-entropy loss function is minimized, as shown in Equation 4:


(4) $$\begin{array}{l} L=-\overset{N}{\underset{i=1}{\sum }}t_{i}\log\left(y_{i}\right) \end{array}$$

where *t*_*i*_ represents the true label and *y*_*i*_ is the predicted probability for each entity class. By minimizing this loss function, the model is trained to improve its performance in recognizing the correct entities.

The integration of LoRA with prompt-based learning further enhances the model's adaptability to specific domains. Prompt-based learning provides explicit task instructions, enabling the model to focus on clearly defined objectives. In the context of diabetes-related entity recognition, the combination of LoRA fine-tuning with prompt-based learning enables the model to accurately identify critical entities, even in resource-constrained scenarios. This integration leverages the contextual guidance provided by prompt-based learning, allowing LoRA's low-rank matrix decomposition to effectively capture domain-specific knowledge, thereby significantly improving the model's performance in complex tasks.

### 3.3 Intent recognition method

The intent recognition module aims to determine the user's query intent and classify it into one of several predefined categories, such as “query disease cause” or “query treatment methods.” Intent recognition plays a crucial role in generating accurate and relevant responses, which directly influences the quality and utility of the system's output. This study employs prompt-based learning using the Qwen2.5 model for intent classification, thereby eliminating the need for additional training phases. The Qwen2.5 model is particularly well-suited for handling complex natural language understanding tasks, especially in the medical domain, where it effectively classifies intent through prompt-based methods (28).

To classify a user query *x* into one of the predefined categories *C*, the Qwen2.5 model utilizes prompt-based learning to identify the most probable intent. Instead of directly maximizing the probability, an attention mechanism is introduced to evaluate the relevance of different parts of the input. The attention score α_*i*_ for each token *x*_*i*_ is computed as illustrated in Equation 5:


(5) $$\begin{array}{l} \alpha_{i}=\frac{\exp\left(e_{i}\right)}{\sum_{j=1}^{n}\exp\left(e_{j}\right)} \end{array}$$

where *e*_*i*_ represents the energy score for token *x*_*i*_, and *n* is the total number of tokens. The attention scores α_*i*_ are used to compute a context vector *c* that summarizes the input sequence, as shown in Equation 6:


(6) $$\begin{array}{l} c=\overset{n}{\underset{i=1}{\sum }}\alpha_{i}x_{i} \end{array}$$

Using the context vector *c*, the model can then determine the intent by computing the probability distribution over all possible categories *C*, as shown in Equation 7:


(7) $$\begin{array}{l} P\left(c|x,\theta \right)=\text{softmax}\left(W_{c}c+b_{c}\right) \end{array}$$

where *W*_*c*_ and *b*_*c*_ are trainable parameters that help map the context vector to the output category space.

During prompt-based classification, the model's objective is to minimize a margin-based ranking loss, which helps distinguish the correct intent category from incorrect ones, as shown in Equation 8:


(8) $$\begin{array}{l} L=\overset{N}{\underset{i=1}{\sum }}\max\left(0,1-f\left(x^{\prime },y_{i}\right)+f\left(x^{\prime },y_{\text{neg}}\right)\right) \end{array}$$

where $f\left(x^{\prime },y_{i}\right)$ is the score for the true category, and $f\left(x^{\prime },y_{\text{neg}}\right)$ is the score for a negative (incorrect) category. This loss encourages the model to assign a higher score to the correct category than to incorrect ones by a margin of at least 1.

The prompt-based approach utilized in this study enhances the model's adaptability by employing specific prompts to guide both classification and answer generation. This strategy is particularly advantageous for intent recognition within the medical domain. By reducing reliance on extensive labeled datasets, it concurrently improves the accuracy of query interpretation. Moreover, the integration of prompt-based learning enables the model to swiftly adapt to various tasks, thereby significantly enhancing its performance in handling complex scenarios.

Table 2 presents the systematic approach employed for intent classification through prompt engineering. This comprehensive strategy ensures complete coverage of potential user intents, ultimately resulting in more precise and contextually appropriate responses. By following the process outlined in Table 2, the model can accurately identify and classify user intents, thereby enabling more robust and context-sensitive responses. This capability is especially critical in the medical domain, where precision and nuance are paramount and can significantly affect patient care and treatment outcomes.

**Table 2:** Systematic intent classification prompt strategy.

**Step**	**Description**
Analyze user query	Analyze the user's question one by one to check if it fits into the nine predefined query categories. Ensure all potential intents are fully considered.
Category matching	For each of the nine predefined categories, determine if it matches the user's question. If a match is found, add the category to the output list.
Identify implicit intent	Consider not only explicit intent but also identify the implicit intent within the user's question, ensuring no important categories are overlooked.
Add to output list	Add all matched categories to the output list to ensure comprehensive coverage of the user's intents, avoiding any omissions.
Thinking process	The prompt model uses a systematic process to identify user intent, ensuring thoroughness and accuracy by matching each category.

## 4 Empirical research

This chapter provides a comprehensive overview of the dataset, experimental setup, model training procedures, evaluation methodologies, and graph construction techniques. The study focuses on two primary tasks—Named Entity Recognition (NER) and Intent Recognition (IR)—which serve as foundational components for constructing knowledge graphs and developing subsequent question-answering systems, particularly within the context of diabetes. Extensive experiments were conducted to evaluate the model's efficacy, demonstrating its potential applicability and broad utility in diabetes-related applications.

### 4.1 Datasets

#### 4.1.1 Named entity recognition dataset

The dataset utilized in this research originates from the DiaKG diabetes literature dataset, which was part of the CCKS2021 task. Provided by the Alibaba Cloud Tianchi Big Data Platform, this dataset comprises 41 consensus documents on diabetes, covering various aspects such as foundational research, clinical studies, medication protocols, case studies, and diagnostic methods. These documents represent the major research trends in diabetes over the past seven years (29). The dataset includes annotations for 22,050 medical entities and 6,890 relationships, spanning 18 entity types and 15 relationship categories.

During dataset construction, data cleaning and segmentation were conducted to eliminate redundancy and ensure data standardization. To enhance model generalizability and enrich the training dataset, a data augmentation strategy was implemented, expanding each original sample into three distinct samples by categorizing the 18 entity types into three groups (30). Specifically, a task-diversified data expansion approach was applied, reconstructing the original data through varied combinations of entity categories and prompts. Each prompt was aligned with a specific target entity schema, resulting in multiple training samples with diverse structures (31). An example demonstrating how each original data point was expanded into three distinct samples is presented in Table 3. Finally, the dataset was partitioned for model training and evaluation purposes. The training set consists of 9,033 samples, while the test set includes 1,491 samples. This partition ensures sufficient data for training and provides an effective basis for evaluating the model's performance in entity recognition tasks.

**Table 3:** Example of a table showing one piece of data expanded into three pieces of data.

**Importation**	**Exports**
Cellulitis fused to form a large abscess, with increased purulent secretions and necrotic tissue, dry gangrene of the foot or few toes, but bone destruction is not yet obvious.	Surgery:[], Location:[“Foot”, “Toe”], Clinical Manifestations:[“Large Abscess”, “Purulent Secretions and Necrotic Tissue”, “Bone Destruction”], Examination Indicators:[], Medication Dosage:[], Examination Methods:[]
Cellulitis fused to form a large abscess, with increased purulent secretions and necrotic tissue, dry gangrene of the foot or few toes, but bone destruction is not yet obvious.	Medication Name:[], Disease Staging and Classification:[], Adverse Reactions:[], Medication Frequency:[], Cause:[], Severity:[]
Cellulitis fused to form a large abscess, with increased purulent secretions and necrotic tissue, dry gangrene of the foot or few toes, but bone destruction is not yet obvious.	Non-Medication Treatment:[], Pathogenesis:[], Examination Indicator Values:[], Medication Methods:[], Duration:[], Diseases:[“Cellulitis,” “Dry Gangrene of Foot or Few Toes”]

#### 4.1.2 Intent recognition dataset

The intent recognition dataset systematically defines nine diabetes-related query intents: querying disease overview, causes, required medications, recommended foods, foods to avoid, necessary tests, symptoms, treatment methods, and concurrent diseases. These intents capture the primary user needs for diabetes-related information, reflecting the complexity of medical knowledge and the diversity of user inquiries.

The data expansion process focused on ensuring the diversity and validity of generated samples. The GPT-4 model was employed to generate numerous high-quality query samples for each intent category, effectively mitigating the patterning issues often associated with manually designed datasets. This approach enriched the dataset's diversity and representativeness. Additionally, manual review and optimization were conducted to ensure the semantic accuracy and reliability of the medical information in each sample.

The constructed dataset provided balanced training samples for the nine intent categories, ensuring comprehensive learning of each intent's distinctive features. The finalized dataset contained 1,066 samples. To facilitate effective model training and testing, the dataset was partitioned in an 80:20 ratio, resulting in 841 training samples and 225 test samples. This partition ensured sufficient data for training while maintaining an adequate number of samples for effective evaluation of model performance in intent recognition tasks. Selected data samples are presented in Table 4.

**Table 4:** Input questions along with their corresponding intent categories.

**Input question**	**Intent category**
Is sitagliptin used for treating diabetes?	[“Query disease overview”, “Query required medication”, “Query treatment methods”]
What dietary precautions should patients with chronic kidney disease take?	[“Query disease overview”, “Query recommended foods”, “Query restricted foods”]
What is OSAHS? What examinations should patients undergo?	[“Query disease overview”, “Query required examinations”]
What symptoms does spinal cord disease cause? What examinations are needed?	[“Query disease overview”, “Query symptoms”, “Query required examinations”]
What is non-alcoholic fatty liver disease? Is it caused by eating too much greasy food?	[“Query disease overview”, “Query causes of disease”]
What are the symptoms of diabetic mononeuropathy? What medication is required for treatment?	[“Query disease overview”, “Query symptoms”, “Query required medication”]

### 4.2 Named entity recognition experiment

#### 4.2.1 Experimental environment and hyperparameter settings

The experiments were conducted on an Ubuntu 20.04 system equipped with an NVIDIA A10 GPU featuring 24 GB of memory. The Baichuan2-13B-Chat model was used as the pre-trained model. The learning rate was set to 3e-5, with three training epochs, a gradient accumulation step of four, and batch sizes of two for training and six for evaluation. The AdamW optimizer was employed, with a maximum input sequence length of 400, a truncation length of 700, and a maximum output length of 300. The Low-Rank Adaptation (LoRA) hyperparameters were configured with a rank of 16, an alpha value of 32, and a dropout rate of 0.1. A summary of the hyperparameter settings is presented in Table 5.

**Table 5:** Hyperparameter settings for entity recognition experiment.

**Hyperparameter**	**Value**
Learning rate	3e-5
Training batch size	2
Gradient accumulation	4
Training epochs	3
Optimizer	AdamW
Rank (LoRA)	16
α value (LoRA)	32
Dropout rate (LoRA)	0.1

The table lists the hyperparameters used in the experiment, along with their respective values.

#### 4.2.2 Experimental results and analysis

The entity recognition experiment compared the performance of several models, including RoBERTa, BiLSTM-CRF, RoBERTa-BiLSTM, RoBERTa-CRF, BERT-BiLSTM-CRF, and LoRA-Prompt-Baichuan2, as detailed in Table 6. Precision, recall, and F1-score were used as the primary evaluation metrics to comprehensively assess model performance.

**Table 6:** Entity recognition experiment results.

**Entity recognition experiment results**			
**Model**	**Precision (%)**	**Recall (%)**	**F1-score (%)**
RoBERTa	66.84	76.32	71.27
BiLSTM-CRF	66.52	64.30	65.39
RoBERTa-BiLSTM	72.76	76.43	74.48
RoBERTa-CRF	73.30	81.16	77.03
BERT-BiLSTM-CRF	80.24	78.90	79.56
LoRA-Prompt-Baichuan2	85.91	80.59	83.17

The table presents the experimental results of various models on entity recognition tasks, using precision, recall, and F1-score as the evaluation metrics.

The baseline model, RoBERTa, achieved an F1-score of 71.27%, whereas the BiLSTM-CRF model obtained a lower F1-score of 65.39%, highlighting the limitations of basic deep learning models in entity recognition. Incorporating CRF or BiLSTM structures into RoBERTa significantly enhanced performance, with RoBERTa-CRF achieving an F1-score of 77.03% and RoBERTa-BiLSTM reaching 74.48%. This suggests that adding sequence dependency mechanisms improves the model's ability to recognize entity boundaries.

The BERT-BiLSTM-CRF model, which integrated BERT's pre-trained features with BiLSTM and CRF, achieved an F1-score of 79.56%, demonstrating strong capabilities in capturing complex semantics and handling long-span entities. These results underscore the effectiveness of combining pre-trained models with sequence-aware structures like CRF and BiLSTM, significantly enhancing the capture of semantic relationships and improving entity recognition performance.

The LoRA-Prompt-Baichuan2 model outperformed all others, achieving a precision of 85.91%, recall of 80.59%, and an F1-score of 83.17%. This model combined Baichuan2's pre-training capabilities with prompt-based learning and LoRA fine-tuning, resulting in significant improvements in entity recognition. Flexible prompt design allowed the model to adapt effectively to specific tasks, while LoRA fine-tuning improved generalization capabilities without significantly increasing the number of parameters. Compared to traditional fine-tuning methods, LoRA-Prompt-Baichuan2 demonstrated clear advantages in handling complex relationships, recognizing long-span entities, and improving overall accuracy.

Overall, the LoRA-Prompt-Baichuan2 model excelled across precision, recall, and F1-score metrics, showcasing substantial potential and efficiency in managing complex entities and diverse tasks. Future research could focus on optimizing prompt design and validating the model's generalization across domain-specific tasks, thereby expanding its applicability and enhancing performance.

### 4.3 Intent recognition experiment

#### 4.3.1 Experimental environment

The experimental setup was meticulously configured to ensure compatibility and efficiency throughout the model training process. ModelScope version 1.14.0 and PyTorch version 2.1.2 were employed to maximize GPU acceleration, utilizing Python 3.10 and CUDA version 12.1. The experiments were conducted on an Ubuntu 22.04 system, chosen for its proven stability and capability to manage computationally intensive tasks effectively.

The Qwen2.5-7B-Instruct model, comprising 7 billion parameters, was selected due to its demonstrated efficacy in handling complex natural language processing (NLP) tasks, particularly intent classification in specialized healthcare domains. The computational infrastructure was optimized to facilitate efficient training and ensure the reproducibility of experimental results, thereby enhancing the reliability and validity of the study's conclusions.

#### 4.3.2 Experimental results and analysis

The experiment evaluated the performance of several models for intent recognition, specifically BiLSTM-attention, BERT, BERT-TextCNN, and Prompt+Qwen2.5, as presented in Table 7. Precision, recall, and F1-score were used as the primary evaluation metrics to comprehensively assess each model.

**Table 7:** Intent recognition experiment results.

**Intent recognition experiment results**			
**Model**	**Precision (%)**	**Recall (%)**	**F1-score (%)**
BiLSTM-attention	64.73	62.87	52.81
BERT	74.70	71.12	72.82
BERT-TextCNN	75.67	72.28	73.88
Prompt-Qwen2.5	88.55	84.65	86.48

The table shows precision, recall, and F1-score for different models.

The BiLSTM-attention model demonstrated moderate performance, achieving a precision of 64.73%, recall of 62.87%, and an F1-score of 52.81%. Despite employing an attention mechanism to emphasize significant sequence information, the model struggled with longer texts and complex semantic relationships due to BiLSTM's inherent limitations. For complex intent recognition tasks, the BiLSTM-attention model underperformed compared to pre-trained language models.

In contrast, the BERT and BERT-TextCNN models demonstrated significantly better performance. BERT, utilizing its deep self-attention mechanisms, exhibited strong semantic understanding, achieving a precision of 74.7%, recall of 71.12%, and an F1-score of 72.82%. The BERT-TextCNN model further enhanced performance by integrating a convolutional neural network (CNN) to effectively extract local features, particularly for phrases or keywords, resulting in a precision of 75.67%, recall of 72.28%, and an F1-score of 73.88%. These results indicate that pre-trained language models, when integrated with downstream architectures, can significantly enhance intent recognition performance.

The Prompt-Qwen2.5 model achieved the highest performance, with a precision of 88.55%, recall of 84.65%, and an F1-score of 86.48%. This model significantly outperformed other models, highlighting the advantages of generative large language models in intent recognition. Notably, the Prompt-Qwen2.5 model bypassed traditional training by using crafted prompts to directly operate on the test set, achieving excellent results. This finding suggests that pre-trained large language models can rapidly adapt to specific tasks through prompt engineering, thereby eliminating the need for additional training and conserving time and computational resources.

Overall, the Prompt-Qwen2.5 model not only surpassed other models in performance but also demonstrated exceptional practicality and flexibility. Its ability to be directly applied to data-driven tasks without further training underscores its efficiency. Future research should focus on optimizing prompt design, investigating its impact on performance, and validating its generalization across diverse tasks to advance intent recognition.

### 4.4 Construction of the diabetes knowledge graph

#### 4.4.1 Knowledge graph conceptual design

A knowledge graph is a directed, labeled graph consisting of nodes and edges. Nodes represent entities (such as diseases and drugs), concepts, or attribute values, while edges denote relationships or attributes between entities (19). The fundamental data structure of a knowledge graph is a triple: (entity1, relationship, entity2), effectively capturing relational information (32).

The construction of the diabetes knowledge graph leverages Neo4j, an efficient graph database specifically designed for storing and querying complex graph data. Neo4j represents information using nodes, relationships, and properties, thereby providing an intuitive depiction of entities and their interconnections, making it particularly well-suited for knowledge graph construction. Furthermore, Cipher, Neo4j's dedicated query language, facilitates efficient graph data manipulation, enabling the formulation of complex queries and the exploration of intricate relationships. Neo4j's capabilities extend to handling large-scale graph data, supporting parallel processing and distributed architectures, and providing a library of graph algorithms for analyzing complex network relationships (33).

#### 4.4.2 Entity and relationship import

The development of the diabetes knowledge graph involves integrating various heterogeneous data sources to form a comprehensive network of diseases, symptoms, medications, and related entities (34). Key attributes such as causes, treatments, and overviews are added to disease nodes to provide detailed information. Neo4j is utilized to store this information efficiently and facilitate the dynamic retrieval of relevant medical data, which can then be incorporated into contextual prompts for natural language models. Neo4j's advanced storage and querying capabilities allow researchers to directly analyze entities and their relationships, significantly enhancing the efficiency of both clinical diagnostics and scientific research (35).

**Figure 3:**
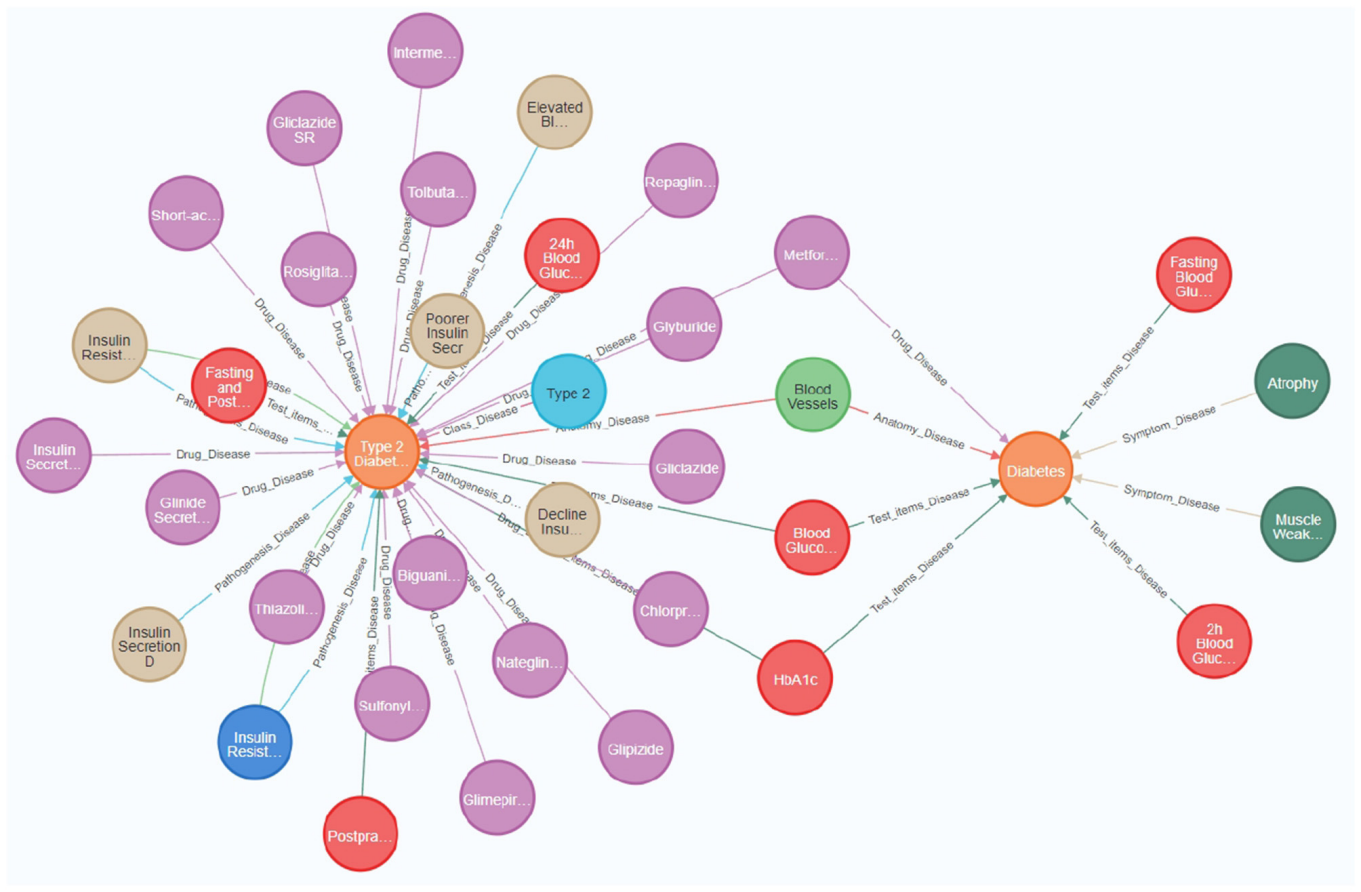
The diabetes knowledge graph offers a comprehensive visualization of relationships among diseases, symptoms, medications, and associated medical entities. Key nodes in the figure represent essential medical concepts, such as “Diabetes,” “Type 2 Diabetes,” “Blood Glucose,” and specific medications like “Metformin” and “Gliclazide.” Relationships between nodes indicate significant medical associations, including the relationship between ”Type 2 Diabetes“ and various drugs used for its treatment, as well as the link between “Diabetes” and its symptoms, such as “Atrophy” and “Muscle Weakness.” The knowledge graph was constructed by integrating heterogeneous data sources, thereby providing a holistic view of diabetes management. It incorporates attributes such as causes, treatments, and diagnostic indicators, which facilitate better understanding and effective medical data retrieval.

Additionally, Neo4j's visualization capabilities significantly improve the usability and interpretability of the knowledge graph, allowing stakeholders to better understand and utilize the complex relational network. Figure 3 provides a visual representation of nodes and relationships within the knowledge graph, illustrating the intricate associations among diseases, symptoms, treatments, and related factors. Such visualization tools are crucial for interpreting interactions within the knowledge graph, thereby facilitating effective utilization of data by researchers and medical professionals (32).

## 5 Intelligent question-answering system development and application

### 5.1 System architecture

**Figure 4:**
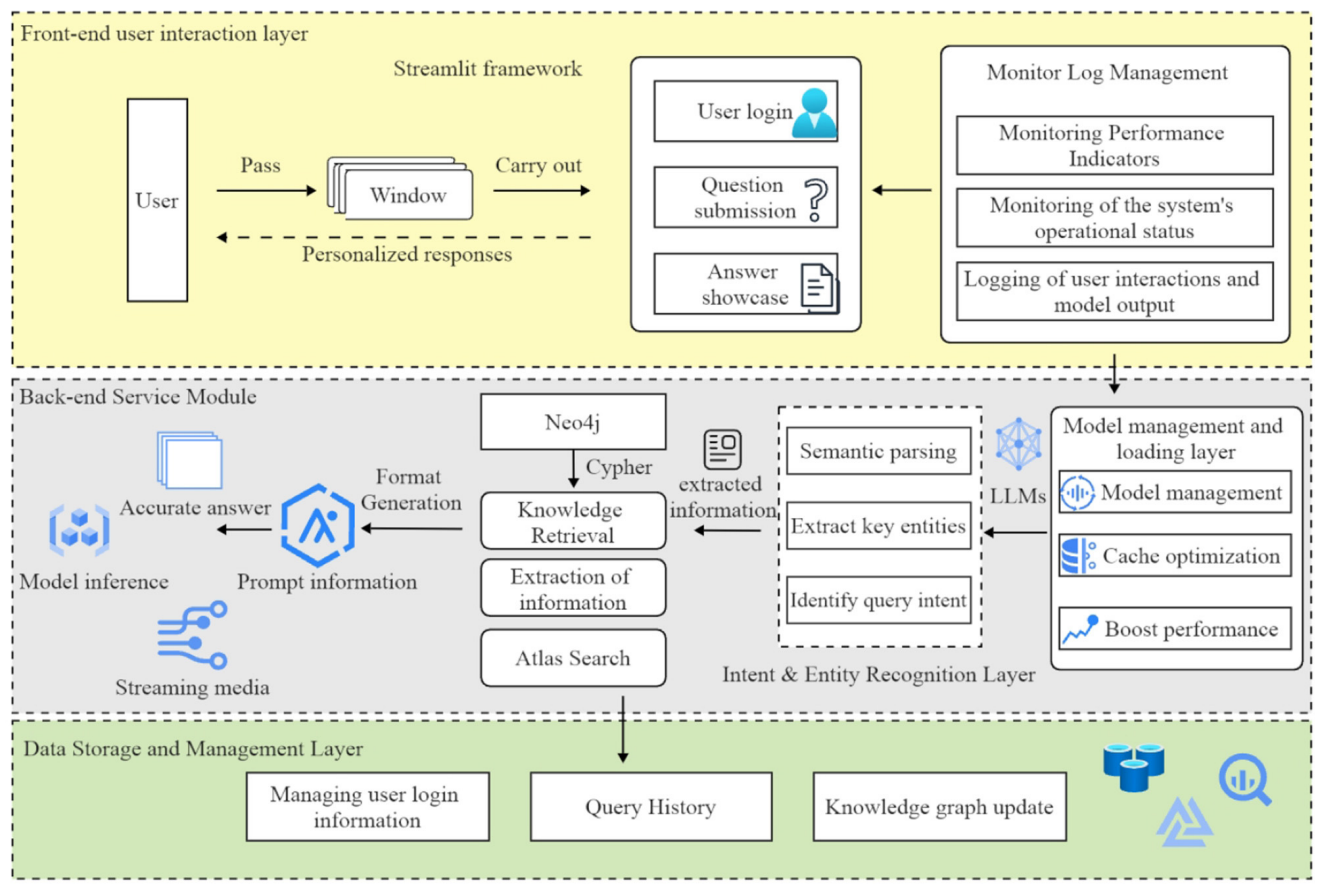
The architecture of the diabetes question-answering (QA) system comprises six primary modules: user interaction, model management, intent recognition, knowledge retrieval, inference, and data management. The arrows represent the flow of information between these modules, effectively illustrating the system's overall workflow.

The architecture of the diabetes intelligent question-answering system consists of six primary modules, ensuring comprehensive functionality and optimal performance (see Figure 4). The first module, the frontend user interaction layer, is implemented using the Streamlit framework, which manages user login, question submission, and answer display through an intuitive interface. Users can submit questions in multiple dialogue windows and receive personalized responses. This frontend layer communicates with both the backend model processing layer and the knowledge graph layer to provide real-time responses.

The model management and loading layer is responsible for loading and managing pre-trained language models. This module uses caching mechanisms to optimize model loading, thereby reducing redundancy and improving responsiveness to user queries.

The intent and entity recognition layer processes user questions to identify their intent and extract key entities. This module utilizes fine-tuned language models to analyze user input, determine the query type (e.g., “disease overview” or “treatment methods”), and identify related entities such as symptoms and medications. The extracted information is then used to generate appropriate knowledge graph queries, thereby ensuring the provision of accurate answers.

The knowledge graph query layer employs Neo4j to store and manage diabetes-related knowledge. This module uses Cipher queries to retrieve relevant information, which is subsequently formatted as prompts for the reasoning model to generate responses.

The inference and answer generation layer employs language models (e.g., Qwen2) to generate precise responses. These models create accurate answers based on prompts obtained from the knowledge graph query layer, with a streaming feature for real-time response viewing. The output is regulated to prevent freeform generation, ensuring consistency and reliability.

The data storage and management layer handles user login information, query history, and knowledge graph updates. This module stores user data in a MySQL database, allowing users to review their query history and receive personalized recommendations. It also manages administrative permissions for maintaining and updating the knowledge graph.

### 5.2 Intelligent question-answering system design

#### 5.2.1 Intelligent question-answering system workflow

The diabetes intelligent question-answering system is a significant application of artificial intelligence in managing diabetes-related knowledge, designed specifically to address health inquiries posed by patients using natural language. This system aims to enhance patient access to diabetes-related information, thereby promoting the dissemination of health knowledge. One major challenge is accurately interpreting patient questions, retrieving relevant information, and generating precise responses.

**Figure 5:**
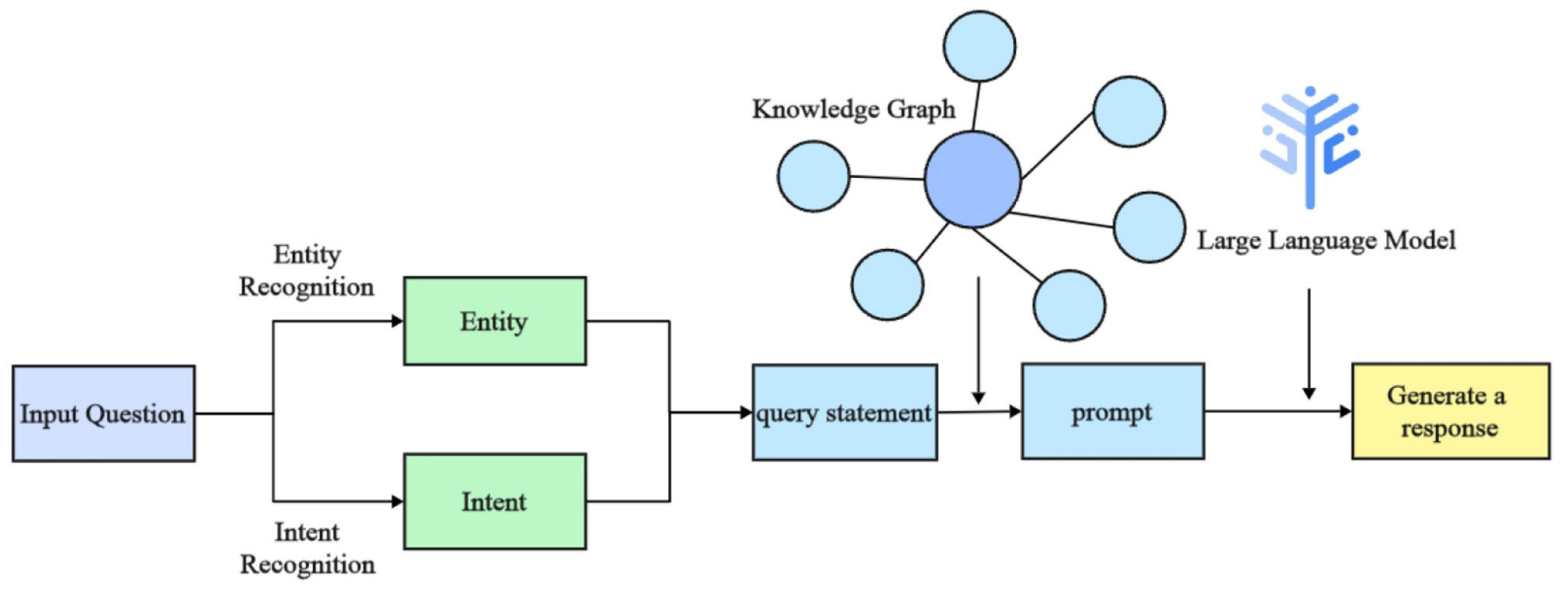
Workflow diagram of the intelligent question-answering (QA) system for diabetes. The diagram outlines the sequential steps from user input to response generation, illustrating key processes such as intent recognition, entity extraction, and the use of a knowledge graph and language models.

Recent advancements in natural language processing (NLP) have significantly improved the system's performance, particularly in intent recognition and named entity recognition (NER). These capabilities allow the system to better understand patient needs, enabling the provision of personalized medical advice and facilitating self-management for diabetes patients. The workflow of the intelligent question-answering system, illustrated in Figure 5, consists of five primary steps:

1. **Question input:** the user submits a diabetes-related question in textual form.
2. **Intent recognition:** the system utilizes a large language model to determine the user's query intent, which may involve understanding diabetes causes, treatment options, medication usage, or complications.
3. **Entity recognition:** the system employs a fine-tuned Baichuan2 model to extract key diabetes-related entities, such as disease and medication names.
4. **Knowledge graph query:** based on the recognized entities and identified intent, the system queries the relevant medical knowledge stored in the Neo4j knowledge graph.
5. **Return result:** the system synthesizes the retrieved information using a large language model to generate a comprehensive response, presented in natural language.

#### 5.2.2 Knowledge graph query

In the intelligent question-answering system, querying the diabetes knowledge graph relies on the combined functionalities of entity and intent recognition. First, the system determines the user's query intent, such as inquiries about symptoms, preventive measures, or treatment options. Subsequently, the system extracts key entities, such as “diabetes,” medications, or symptoms, from the user's input.

The identified entities are then embedded into Cipher queries to search for relevant nodes and relationships within the knowledge graph, thereby enabling the retrieval of precise information. For example, if a user asks, “How can diabetes complications be prevented?”, the system identifies the user's intent as seeking preventive measures for diabetes complications and extracts “diabetes complications” as the core entity. Using this information, the system generates a Cipher query to retrieve data from the knowledge graph. The results may include recommendations such as “control blood sugar levels,” “regular check-ups,” and “maintain a healthy diet.” The system then synthesizes these recommendations into a concise response, such as: “To prevent diabetes complications, it is recommended to control blood sugar levels, conduct regular foot check-ups, and follow a physician-guided medication plan.”

This approach ensures that the system provides accurate answers while leveraging the knowledge graph to deliver comprehensive health advice, ultimately assisting users in managing diabetes effectively.

#### 5.2.3 Prompt generation and response

With the increasing adoption of generative pre-trained language models, prompt generation has emerged as a crucial technique for enhancing model performance. Prompt generation involves designing structured input prompts to help the model understand task objectives and contextualize the output, thereby producing high-quality responses. Depending on the generation method, prompt generation can be classified into template-based, automated, and hybrid approaches. Core techniques include natural language parsing, context integration, and dynamic optimization to ensure that prompts effectively convey relevant information in complex tasks. Primary methods for evaluating and optimizing prompt generation include manual evaluation, automated evaluation, and iterative refinement. Prompt generation has demonstrated significant potential in tasks such as question answering and text generation, with ongoing research focusing on improving the flexibility, stability, and accuracy of generated prompts.

In the diabetes intelligent question-answering system, prompt generation plays a pivotal role in guiding the generative language model (e.g., Qwen2 or Chatglm3) to produce accurate medical responses. The prompt generation process involves parsing user input via intent recognition to determine the user's query intent. Based on the recognized intent and named entity recognition (NER) results, the system retrieves relevant medical information from the Neo4j knowledge graph, dynamically incorporating this information into the prompt to provide essential background context. During prompt generation, the system explicitly sets task instructions (e.g., “You are a diabetes question-answering assistant”) and imposes strict response constraints (e.g., “Do not provide speculative answers”) to ensure that the model's output is well-grounded in the knowledge base.

The finalized prompt is then submitted to the generative language model, which generates precise medical responses. This hybrid strategy, combining template-based and dynamic prompt generation, enhances the system's performance in medical question-answering tasks by ensuring that the generated responses are professional, accurate, and consistent.

### 5.3 Application interface

**Figure 6:**
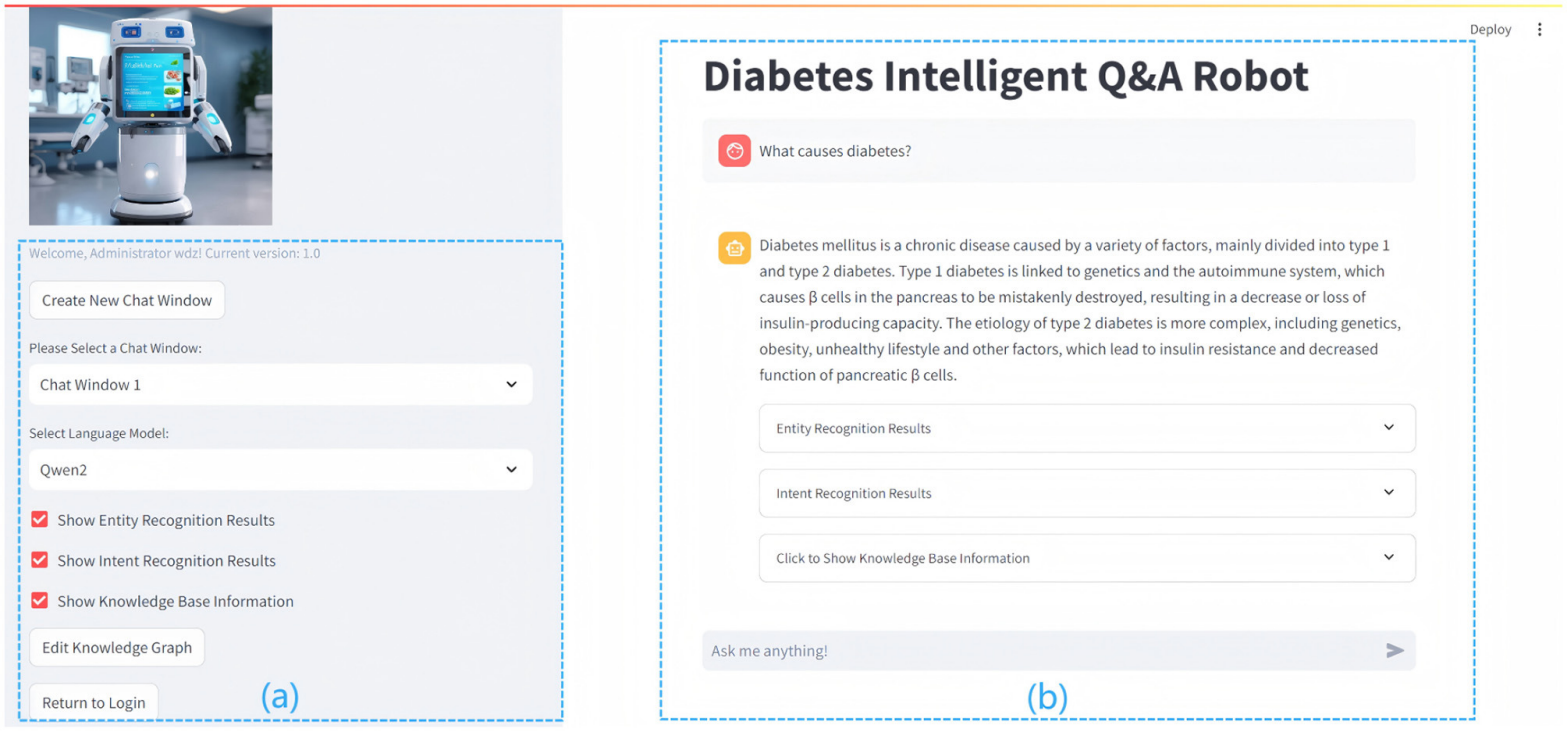
The figure details the interface and functionality of the Diabetes Question and Answer (QA) system. The left section **(A)** shows the functional area with configurable options, while the right section **(B)** presents the user interface, displaying diabetes-related queries and system-generated responses.

The intelligent question-answering interface, depicted in Figure 6, serves as the primary platform for user interaction with the system. Users input diabetes-related questions, which are subsequently processed by the system using natural language processing (NLP) techniques such as named entity recognition (NER) and intent recognition. NER extracts key entities, including disease names and symptoms, from the user's input, while intent recognition discerns the user's specific needs to provide accurate responses.

(a) On the left side of Figure 6, the functional area offers a range of operational options, including the creation and management of chat windows, the selection of language models, and the activation of features such as entity recognition, intent recognition, knowledge base display, and knowledge graph editing. These tools facilitate efficient interaction with the system, ensuring that it can accommodate various user needs. (b) On the right side of Figure 6 is the user interaction interface, which displays diabetes-related queries and system-generated responses, illustrating the system's advanced natural language processing capabilities. The interface incorporates entity recognition, intent recognition, and knowledge-base-driven response generation, enabling users to obtain accurate and relevant information, while the input box at the bottom allows users to submit queries, such as “What are the common symptoms of diabetes?” or “How can blood sugar be managed?” for suggestions on symptoms and treatments. The interface prioritizes user experience through a clear layout and soft color scheme, facilitating seamless interaction and aiding users in managing their health information effectively.

The QA display is central to user interaction. The system processes user inputs and employs NLP techniques to generate accurate, real-time responses. For instance, if a user asks, “What causes diabetes?”, the system preprocesses the question, extracting key content such as “diabetes” via NER, and determines the intent, such as “disease overview and causes.” After interpreting the user's needs, the system generates a query to retrieve relevant information from the knowledge graph. The system then synthesizes the retrieved knowledge with outputs from NLP models (e.g., Qwen2) to generate precise, natural responses, delivering authoritative and comprehensible information to enhance the user experience.

## 6 Discussion

### 6.1 Research objectives and contributions

The primary objective of this research was to develop an intelligent question-answering system specifically designed for diabetes management, incorporating Named Entity Recognition (NER), Intent Recognition (IR), and an extensive knowledge graph (KG). This methodology addresses the inherent limitations of traditional medical question-answering systems, such as inadequate personalization and limited semantic comprehension in medical contexts. Existing systems, such as MySugr or BlueLoop, focus primarily on glucose monitoring but fail to account for the diverse and complex needs of patients or to dynamically update their knowledge base.

In contrast, this research adopts a hybrid architecture combining large language models (LLMs) with a Neo4j-based knowledge graph to enhance the precision and relevance of responses (36). By integrating real-time data from the knowledge graph, the proposed system can deliver both explicit and implicit responses, significantly improving user experience and increasing applicability in clinical environments. This approach allows for dynamic updates to the knowledge base, offering more personalized and context-aware answers to complex medical queries.

### 6.2 Innovation in prompt-based learning and Low-Rank Adaptation (LoRA)

A key innovation of this study is the application of prompt-based learning alongside Low-Rank Adaptation (LoRA) to enhance both entity and intent recognition. Traditional models, such as BiLSTM, BERT, and RoBERTa, generally require extensive fine-tuning for domain adaptation, necessitating substantial computational resources and large amounts of labeled data. In contrast, integrating prompt-based learning with LoRA provides a lightweight and flexible approach capable of effective adaptation, even in low-resource settings (37).

The LoRA fine-tuning mechanism significantly reduces the number of trainable parameters while preserving high accuracy in identifying long-span entities'an area that has traditionally posed challenges for conventional methods due to complex linguistic structures. Additionally, using Qwen2.5 for intent classification enables direct prompt-based categorization without the need for additional fine-tuning, offering a novel solution compared to traditional models. These innovations are particularly beneficial in low-resource environments, demonstrating promising results in diabetic patient data processing (38).

### 6.3 Knowledge graph construction and integration

Constructing the knowledge graph and integrating it with the intelligent question-answering system represents a significant contribution of this study. Unlike traditional healthcare knowledge graphs, which primarily depict static relationships between entities, this study employs a dynamic Neo4j-based graph that facilitates real-time retrieval and supports complex queries. This dynamic approach allows for the continuous adaptation and maintenance of healthcare data, greatly enhancing the system's practical utility (39). By integrating large language models such as Baichuan2 and Qwen2.5, the system is able to handle complex medical inquiries and provide contextually enriched responses (40).

While the knowledge graph enhances the system's ability to deliver accurate and relevant answers, challenges remain in maintaining the real-time relevance of the medical data it contains (41). Future work should focus on automating the updating process for healthcare data, ensuring that the graph reflects the latest research, treatment protocols, and medical knowledge (42).

### 6.4 Prompt generation strategy

The prompt generation strategy employed in this study integrates both template-based and dynamic generation techniques, ensuring that responses are grounded in precise and comprehensive medical knowledge. This methodology departs from traditional systems that rely exclusively on pre-designed templates, thus overcoming the limitations associated with rigid, non-adaptive responses (43). The proposed system's ability to dynamically generate relevant prompts allows it to address diverse medical queries, making it more versatile than existing systems.

Although the system has shown promising results in entity recognition and intent classification, its usability in real-world healthcare environments has not been extensively tested. The system has primarily been evaluated in controlled, experimental setups. However, to validate its true potential, future work should focus on conducting usability testing with healthcare professionals and patients in real-world clinical settings (44). This will involve assessing the system's ease of use, its ability to integrate seamlessly with existing clinical workflows, and its capacity to meet the diverse needs of users in a healthcare context.

Feedback from healthcare providers and diabetic patients will be essential to refine and improve the system. Formal feedback collection from patients regarding their experience with the system is still pending, which limits our understanding of how effectively the system addresses their needs. Future work should prioritize the collection of patient feedback to further tailor the system and enhance its relevance to the patient community. Engaging diabetic patients through usability studies or pilot programs will provide valuable insights into system optimization, ensuring that it delivers actionable and relevant health information that meets their specific requirements (45).

### 6.5 Ethical and privacy considerations

This study's handling of sensitive medical data required meticulous attention to ethical and privacy concerns, particularly given the inherently personal nature of diabetes-related information. For the Named Entity Recognition (NER) task, we employed the publicly available and anonymized CCKS2021 diabetes dataset, which adheres to stringent ethical guidelines, ensuring that no personally identifiable information is involved. Access to the dataset was obtained by the terms of use stipulated by its creators, and it complies with international privacy standards, including the General Data Protection Regulation (GDPR).

For the Intent Recognition (IR) task, a custom dataset was synthetically generated using OpenAI's GPT-4 model, which avoids using real patient data and significantly mitigates privacy risks. The synthetic dataset underwent rigorous review to ensure its clinical relevance and ethical integrity, reinforcing our commitment to patient privacy while facilitating effective model training.

While anonymized datasets effectively preserve privacy, they may limit the ability to provide highly personalized responses in real-world applications. To address this limitation, future research will investigate advanced privacy-preserving techniques, such as differential privacy and federated learning, to enhance data security without compromising system performance. Ethical oversight was integral throughout this study, with all research activities approved by the relevant institutional ethics committee. As the system advances toward real-world testing and deployment, collaboration with healthcare institutions will prioritize adherence to ethical and legal standards, ensuring the responsible application of artificial intelligence in diabetes management.

### 6.6 Future research directions

The proposed system demonstrates significant advancements in personalized healthcare, yet several key challenges remain that require further attention in future research.

A major challenge is adapting the system to address complex patient scenarios, such as multi-morbidity, where patients suffer from multiple co-existing conditions. While the current model has been optimized for diabetes-related queries, it has not yet been tested in multi-morbidity contexts. Future research will focus on extending the system's capabilities to process and integrate data from multiple diseases simultaneously. This will involve developing models capable of understanding and managing the interactions between co-existing conditions, thereby enabling the system to provide more relevant and personalized care to patients with diverse health profiles. Additionally, ensuring the system's continued relevance as new research, treatment protocols, and patient-specific data emerge is crucial. Future work will focus on automating the real-time updating process of the knowledge graph to maintain its accuracy and timeliness, incorporating advanced text mining and data extraction techniques to reduce manual effort and ensure consistent, evidence-based recommendations. Addressing both multi-morbidity and knowledge graph updates is essential for improving the system's clinical applicability and adaptability to evolving medical knowledge (46, 47).

Although the system has demonstrated effectiveness in controlled environments, its deployment in real-world clinical settings presents several challenges. Future research will prioritize usability testing in collaboration with healthcare professionals and patients, evaluating its integration with Clinical Decision Support Systems (CDSS) and Electronic Health Records (EHRs), as well as its ability to handle large volumes of real-time patient queries. Real-world testing will assess scalability, usability, and accuracy in clinical conditions. Collaborations with healthcare institutions will be essential to refine the system and ensure its seamless integration into existing clinical workflows (48).

## 7 Conclusions

This paper addresses the complexities of information retrieval in diabetes management through the development of an intelligent question-answering system that integrates large language models with knowledge graphs. The study demonstrates that integrating the Baichuan2 model with Low-Rank Adaptation (LoRA) technology and prompt-based learning significantly enhances the accuracy of named entity recognition (NER) and intent recognition, thereby augmenting the system's capacity to provide personalized responses and support medical reasoning (26). Experimental results indicate that the system achieved high precision in both diabetes entity and intent recognition tasks, validating the potential of integrating large language models with knowledge graphs for medical applications (49).

Despite these advancements, several limitations remain. The model's performance is highly dependent on the quality of input data, and additional fine-tuning may be necessary for deployment in other medical domains. Additionally, the reliance on predefined prompts limits the system's adaptability to complex patient scenarios, such as those involving multi-morbidity. The challenge of maintaining an up-to-date knowledge graph remains, limiting real-time accuracy as new medical knowledge and treatment protocols emerge (50).

Future research will address these challenges by extending the system's domain applicability to handle multi-morbidity, automating the process of knowledge graph updates, and conducting real-world testing to evaluate integration with Clinical Decision Support Systems (CDSS) and Electronic Health Records (EHRs). These directions are expected to enhance the system's clinical applicability, ultimately positioning it as a valuable tool for personalized healthcare management across diverse medical domains (51, 52).

## Data Availability

The raw data supporting the conclusions of this article will be made available by the authors, without undue reservation.
